# Exploring Italian Consumers’ Perceptions of Cultivated Meat: Barriers, Drivers, and Future Prospects

**DOI:** 10.3390/nu17193061

**Published:** 2025-09-25

**Authors:** Marcello Stanco, Anna Uliano, Concetta Nazzaro

**Affiliations:** Department of Law, Economics, Management and Quantitative Methods, University of Sannio, 82100 Benevento, Italy; mstanco@unisannio.it (M.S.); cnazzaro@unisannio.it (C.N.)

**Keywords:** consumer behavior, food innovation, alternative food, logistic regression

## Abstract

**Background/Objectives:** The increasing global population and rising demand for protein-rich foods present significant challenges for the agri-food system. Cultivated meat, produced through cellular agriculture, is emerging as a promising alternative to traditional livestock farming, offering potential environmental and ethical benefits. However, its adoption remains controversial due to concerns about sustainability, safety, and cultural acceptance. This study investigates Italian consumers’ perceptions, knowledge, and willingness to purchase cultivated meat, considering psychological, demographic, and social factors. **Methods:** A structured online survey was conducted involving 437 Italian meat consumers, integrating established psychometric scales to assess key attitudes. Logistic regression analysis was applied to identify determinants of consumer acceptance. **Results:** Findings reveal that while awareness of cultivated meat is relatively high (81.92%), willingness to purchase it is low, at just 35.47%. The main motivations for interest are environmental sustainability (54.61%) and innovation appeal (25.00%), while the primary barriers are health concerns (31.58%) and doubts about production processes (34.59%). The results also show that food neophobia, environmental awareness, and inclination toward food innovation significantly influence purchasing decisions. Additionally, demographic factors, such as age, gender, income, and household size, play a crucial role. **Conclusions:** This study provides insights into consumer behavior toward food innovations, informing policymakers and industry stakeholders on strategies to enhance acceptance and promote sustainable food alternatives.

## 1. Introduction

The latest United Nations (UN) projections suggest that the world population could grow to around 8.5 billion in 2030 and exceed 9.7 billion in 2050 [1]. The latter represents a significant challenge for the global agri-food system. Indeed, to feed the world population, food production will need to increase by 70% [2]. In addition, the growth in economic possibilities of large parts of the population, and the globalization of consumption habits, are driving demand for protein-rich foods [3] and, more deeply, for meat, both for its nutritional value and the status associated with it [4]. Indeed, over the past 50 years, global demand for meat has tripled, reaching 340 million tons in 2018 [5,6]. However, livestock farming has become unsustainable, being responsible for approximately 14.5% of all human-induced greenhouse gas emissions and extensive land and water use, contributing to deforestation and biodiversity loss [7].

Considering these challenges, many countries are experimenting with innovative approaches to produce meat in a sustainable way [8,9]. Among these innovations, cultivated meat has emerged as one of the most promising technologies in the food industry. Also known as cultured or cell-based meat, cultivated meat is produced through cellular agriculture, a process that involves the ex vivo culture of animal cells obtained via biopsy. These cells are then grown in bioreactors under controlled conditions, applying bioengineering and tissue engineering principles to create food products [10,11].

Although cultivated meat is still in its early stages of development, and it is still expensive to produce, it holds significant potential to contribute to meeting the global demand for protein-rich foods. It is estimated, in fact, that up to 8000 kg of meat can be produced from a single stem cell [12,13]. Furthermore, this type of meat offers numerous potential benefits, including a significant reduction in the environmental impact of traditional farming practices, decreased greenhouse gas emissions, and improved animal welfare conditions [14,15,16]. Moreover, cultivated meat could help address issues of natural resource scarcity by requiring significantly less water and land than conventional meat production [17].

However, alongside these potential benefits, a critical debate has emerged regarding cultivated foods, raising concerns about their sustainability and safety. Some studies suggest that cultivated meat could have a greater environmental impact, mainly due to the energy-intensive and chemical processes involved in its production, which could lead to new types of pollution or inefficient resource use [14,16,18]. Furthermore, concerns have been raised regarding potential human health risks associated with using growth factors or other substances in producing cultivated meat [15]. These debates highlight the complexity of adopting this technology globally and the need for further research to clarify its benefits and risks.

Some countries have already approved cultured meat for human consumption (e.g., Israel, the United States, and Singapore), while in Europe, no authorization has been granted to date. The first European country to oppose the marketing of cultured meat was Italy, both because of the uncertainties mentioned and its potential impact on livestock farmers. In addition to Italy, several other European countries, including France, Greece, and Romania, have expressed their concerns, asking the European Commission to consider not only the opinion of the European Food Safety Authority (EFSA) but also to evaluate the potential socio-economic impacts before any marketing authorization under the Novel Food Regulation (EU) 2015/2283 [19,20].

Meanwhile, to guarantee greater safety and transparency, the EFSA has published new, clear, and timely guidelines for the approval of “novel foods”, which aspiring producers of cultured meat will have to follow when seeking marketing authorization.

The increasing demand for sustainable solutions in food production, coupled with the first requests for authorization, may soon lead to an evolution of the European regulatory framework. In the coming years, this could lead to the approval of cultured meat-based foods for marketing within Europe. From the consumers’ perspective, despite the potential environmental and health benefits, acceptance of cultivated meat remains a complex issue, influenced by various psychological, social, and demographic factors. In countries like Italy, where traditional food policies emphasize respect for gastronomic culture and local culinary heritage, these factors may influence the acceptance of cultivated meat among Italian consumers [21]. Italians generally prioritize fresh, high-quality ingredients and have a strong attachment to traditional meat products such as cured meats and regional specialties. Moreover, the Mediterranean diet, which is widely followed in Italy, emphasizes a balanced intake of plant-based foods, fish, and moderate meat consumption, potentially shaping consumer perceptions of alternative protein sources, including cultivated meat [22,23,24].

Given these cultural and dietary influences, consumer attitudes toward cultivated meat can be particularly complex and multifaceted. Previous studies that involved European consumers have shown that attitudes toward food innovations, such as cultivated meat, are strongly influenced by both sociodemographic (i.e., age, education, and income) and psychological factors, such as food neophobia, ethical concerns, and perceived risks related to food safety [25,26,27,28,29]. Adopting cultivated meat also involves addressing uncertainties about its long-term implications for human health and the environment, which remain central to ongoing scientific and public debates. While its potential to reduce environmental impact and enhance sustainability is clear, these discussions highlight the need for a deeper understanding of the factors that shape consumer attitudes and behavior.

Accordingly, this study seeks to explore Italian consumer perceptions and knowledge of cultivated meat and their willingness to purchase it. Given the evolving landscape of alternative proteins and the limited contributions in the literature on this topic within the Italian context, this study aims to provide new insights into the factors influencing consumer acceptance. Specifically, it investigates how psychological, demographic, and social variables influence consumer behavior. Using a logistic regression model, the study will assess the impact of each factor on consumers’ purchasing decisions regarding cultivated meat.

The research questions guiding this analysis are as follows:

RQ1: What are consumers’ perceptions and knowledge regarding cultivated meat?

RQ2: What are the factors that influence consumers’ willingness to purchase cultivated meat?

This paper is organized as follows. The next section outlines the methodology adopted, including the data-gathering and analysis processes. Section 3 presents and interprets the findings concerning the research questions, highlighting the key factors influencing consumer attitudes and behavior regarding cultivated meat. Finally, the last section discusses the main insights derived from this study, acknowledges its limitations and implications, and suggests directions for future research.

## 2. Materials and Methods

### 2.1. Data Gathering

To achieve the research objectives, a web-based structured questionnaire was administered to a convenience sample of Italian meat consumers. Data collection took place from August 2024 to November 2024. Participation in the survey was entirely voluntary and anonymous, with an estimated completion time of approximately ten minutes. Before participation, respondents provided informed consent, receiving detailed information about this study’s objectives and the handling of personal data, in accordance with applicable privacy and research ethics regulations.

The questionnaire was divided into five main sections to gather specific information regarding consumer attitudes and perceptions of cultivated meat. The first section explored general meat consumption and purchasing habits. The second section focused on awareness of cultivated foods, prompting participants to associate the term “cultivated food” with up to three words. This was performed with the aim of constructing a word cloud: a visual representation of text data, typically used to depict the most frequent words within a given dataset [30].

The third section specifically addressed cultivated meat, providing a clear definition and examples to ensure participants understood the concept. The survey then assessed respondents’ willingness to purchase cultivated meat, analyzing the reasons for acceptance or resistance. The fourth section utilized several established psycho-attitudinal scales to assess key psychological factors influencing consumer perceptions and behaviors toward food innovations. The Abbreviated Food Technology Neophobia (AFTN) scale was used to measure food neophobia, which refers to the reluctance or fear of adopting new food technologies [31]. This scale provides an indicator of consumers’ openness to novel food products, such as cultivated meat, with higher scores suggesting greater resistance to accepting food innovations. The Green Consumer Value (GCV) scale was employed to assess how environmental sensitivity influences purchasing decisions [32]. This scale is particularly relevant in the context of cultivated meat, as it is marketed as having a smaller ecological footprint compared with conventional meat. The Health Consciousness (HC) scale measured consumer concern about health-related aspects of food consumption, a critical factor in the case of cultivated meat, as perceived health benefits or risks can influence acceptance [33]. The Ethical Food Choice Motives (EFCM) scale evaluated the importance of ethical considerations in food choices, such as animal welfare and sustainability, which are often associated with cultivated meat [34]. Finally, the Consumer Novelty Seeking (CNS) scale captured the tendency of consumers to seek out new and innovative food products [35]. Higher scores on this scale indicate a greater openness to trying novel food innovations like cultivated meat.

These scales were all measured using 7-point Likert scales, allowing for a nuanced analysis of respondents’ attitudes and beliefs. The 7-point Likert scale is commonly used in social science research as it provides a balanced range of response options, facilitating the capture of a range of attitudes, from strong agreement to strong disagreement [36].

Finally, the fifth section of the questionnaire gathered socio-demographic information, including age, gender, level of education, income, and household size. These data were essential for examining how different demographic groups might vary in their attitudes and perceptions of cultivated meat.

### 2.2. Data Analysis

To comprehensively analyze the data, descriptive statistics were first conducted for all variables to characterize the sample and their purchasing and consumption habits regarding meat.

A word cloud analysis was conducted based on the open-ended responses to the question regarding “cultivated food”. This analysis allowed for the identification of the most frequent words and associations related to cultivated food, providing insights into how participants mentally conceptualized this food innovation [30]. The use of a word cloud methodology is valuable as it allows for the identification of dominant associations and cognitive frameworks among respondents, providing insight into public perceptions of cultivated foods. By presenting the most frequent terms in larger fonts, the word cloud visually prioritizes the collective connotations and emotional responses surrounding the concept of cultivated food. This method offers the advantage of easily capturing complex, qualitative data from open-ended responses in an intuitive, accessible format [37,38].

Furthermore, for each psychometric scale, a composite variable was created by calculating the mean score of the respective items. These composite variables were used to assess key psychological factors influencing consumer acceptance of cultivated meat. Each scale was measured using a 7-point Likert scale, and the mean scores represented a summary of the respondents’ attitudes toward food innovation, environmental concerns, health consciousness, ethical considerations, and openness to novelty.

Finally, a logistic regression model was applied to investigate the factors influencing the willingness to purchase cultivated meat. Logistic regression is commonly employed in consumer behavior studies, particularly in the food sector, due to its ability to analyze binary dependent variables, such as the intention to purchase or the acceptance of new food technologies [39,40,41,42,43]. In this study, the dependent binary variable was the propensity to purchase cultivated meat, while the independent variables included the psychometric scales (e.g., AFTN, GCV, HC, EFCM, and CNS), socio-demographic characteristics, and variables related to general meat consumption habits and knowledge of cultivated meat. In particular, the logistic regression model can be represented by the following equation:

logit (P) = β_0_ + β_1_X_1_ + β_2_X_2_ + ⋯ + β_k_X_k_

where logit (P) is the log-odds of the probability P, which represents the likelihood of purchasing cultivated meat (a binary outcome, 1 for yes and 0 for no); β_0_ is the intercept term, representing the log-odds of purchasing cultivated meat when all independent variables are equal to zero; and β_1_, β_2_, ⋯, β_k_ are the regression coefficients corresponding to each independent variable X_1_, X_2_, ⋯, X_k_. These coefficients represent the effect of each independent variable on the log-odds of purchasing cultivated meat. A positive coefficient indicates an increased likelihood of purchasing, while a negative coefficient suggests a decreased likelihood; X_1_, X_2_, and X_k_ represent the independent variables in the model, such as psychometric scales (e.g., AFTN, GCV, HC, EFCM, and CNS), socio-demographic factors (e.g., age, gender, and income), and variables related to meat consumption habits and knowledge of cultivated meat. By estimating the coefficients β_1_, β_2_, ⋯, β_k,_ the model provides insights into which variables have the strongest influence on consumer decisions.

## 3. Results

### 3.1. Sample Description

The study sample consisted of 437 respondents, with an average age of 33.74 years (±13.02), ranging from 18 to 86 years (Table 1). The gender distribution was balanced, with 50.34% identifying as female and 49.66% as male. Household size averaged 3.66 members (±1.06), reflecting a diverse range of living arrangements.

In terms of geographical distribution, most participants (84.67%) resided in Southern Italy and the Islands, while smaller proportions were in the North (11.47%) and Central Italy (3.89%). This distribution highlights a predominantly southern demographic representation in the sample. Employment status varied, with most participants employed as workers (44.62%) or self-employed professionals (13.96%). Students constituted a significant subgroup (32.95%), while smaller percentages were housewives/husbands (2.06%), unemployed (2.97%), or pensioners (1.14%). Education levels revealed that 45.08% of participants held a university degree, and an additional 10.53% had obtained a master’s degree or PhD. High school graduates represented 40.73% of the sample, while 3.66% had only completed primary or secondary school.

Income levels were relatively modest, with the largest group (46.68%) reporting annual earnings between EUR 15,000 and EUR 30,000, followed by 21.97% earning up to EUR 15,000. Higher-income brackets were less common, with 20.37% earning between EUR 31,000 and EUR 45,000, 6.64% between EUR 46,000 and EUR 60,000, and only 4.35% exceeding EUR 60,000 annually.

### 3.2. Meat Consumption and Purchasing Habits

Respondents stated a strong preference for beef (95.4%) and pork (87.4%) (Table 2). Most respondents consume at least two portions of meat weekly, with 42.1% exceeding this threshold.

The purchasing patterns reveal a preference for two distinct, yet familiar, outlets: butcher shops (58.58%) and supermarkets (38.9%). While both channels are used for purchasing meat, the minimal reliance on organic stores (0.92%) or direct purchases from farmers (0.92%) suggests limited consumer engagement with alternative or traceable supply chains. 

### 3.3. Cultivated Meat: Awareness and Willingness to Purchase

Awareness of cultivated meat is relatively high (81.92%), yet a deeper understanding of its production processes remains limited, with only 8.24% claiming strong familiarity with methods such as biotechnology and microbial fermentation (Table 3).

This limited understanding contributes to a relatively low willingness to purchase cultivated meat, with only 35.47% of respondents indicating they would buy it (Table 4).

The motivations for purchasing cultivated meat were mainly driven by a belief in its environmental sustainability (54.61%) and the product’s innovative appeal (25.00%). Conversely, the main reasons for the lack of interest were significant health concerns (31.58%) and doubts about the production process (34.59%). 

### 3.4. Results of Logistic Regression

The logistic regression analysis (Table 5) identifies key factors affecting consumers’ likelihood of purchasing cultivated meat. The results show that fear of adopting new food technologies (AFTNs) has a significant negative effect, with a higher level of skepticism reducing the likelihood of purchase. Conversely, GCV and cultivated meat awareness positively influence purchase likelihood. Income, age, gender, and household size also play significant roles, with higher income increasing the likelihood of purchase, while being male, being older, and living in a larger household all decrease it.

The odds ratio analysis (Table 6) provides a more intuitive understanding of the relationships identified in the logistic regression. An odds ratio (OR) quantifies how a one-unit change in a predictor variable affects the odds of an outcome occurring, holding all other variables constant. An OR greater than 1 indicates that the predictor increases the odds of the outcome, while an OR less than 1 suggests it decreases the odds [44]. 

## 4. Discussion

This study’s findings reveal a complex landscape of perceptions, influenced by psychological, demographic, and behavioral factors. The data indicate a preference for conventional meat products, with 95.4% of respondents consuming beef and 87.4% pork, and a reliance on traditional purchasing channels such as butcher shops and supermarkets, underscoring the significant cultural and habitual role that conventional meat holds in the Italian diet, a trend observed in other European countries with strong culinary traditions [45,46,47]. The minimal reliance on organic stores (0.92%) or direct purchases from farmers (0.92%) suggests a limited consumer engagement with alternative or traceable supply chains [48,49,50,51]. This may indicate a lack of accessibility, awareness, or perceived cost barriers associated with such sources.

The divergence between the high awareness of cultivated meat and the low willingness to purchase points to a considerable perception gap. This discrepancy is driven by the limited understanding of the production process, as only 8.24% of respondents expressed high confidence in their knowledge. The qualitative data, which revealed negative word associations like “laboratory” and “artificial,” underscore a general public skepticism rooted in a lack of familiarity and a perception of the product as unnatural. This aligns with previous studies demonstrating that consumer acceptance of novel foods is often inversely related to their perceived “naturalness” and is heavily influenced by a lack of knowledge and trust in the underlying technology [52,53,54].

The motivations for willingness to purchase underscore the perceived environmental advantages of cultivated meat (54.61%) and its innovative appeal (25.00%). This aligns with broader trends where sustainability and novelty drive interest in food innovations [55,56,57]. However, the substantial proportion of respondents citing health concerns (31.58%) and distrust in production processes (34.59%) reflects significant skepticism toward cultivated meat, as also pointed out in the studies of Heiskanen and Ryynänen [58] and Szejda et al. [59]. The prominence of health concerns further reinforces the critical role that health-related factors and safety perceptions play in shaping consumer food choices [60].

The logistic regression analysis provided critical insights into the specific determinants of adoption. The strong negative effect of the AFTN [31] on purchase likelihood is a powerful indicator of consumer neophobia. With an odds ratio of 0.277, a rise in skepticism dramatically reduces the odds of purchase, a finding consistent with prior research on consumer resistance to lab-grown products and genetically modified organisms, where psychological barriers often outweigh objective information [61,62]. 

Conversely, GCV [32] is a significant positive predictor of purchase likelihood (OR = 1.697), which is a key strategic insight. This confirms that pro-environmental attitudes and the desire for sustainable choices are motivators for consumers, a conclusion supported by a growing number of studies identifying sustainability as a primary driver of interest in alternative proteins [63,64,65]. 

The demographic profile of the likely early adopter (i.e., a younger, higher-income woman in a smaller household) further supports a targeted market entry strategy. Younger generations typically exhibit greater openness to food innovations and a higher propensity for sustainable consumption [66,67,68,69]. The role of income may also suggest that cultivated meat will initially be a premium product. The non-significant findings for variables such as health concerns and education, contrary to some existing literature [70,71,72,73], may be unique to this Italian sample, suggesting a need for more nuanced qualitative research to explore these factors.

### 4.1. Implications for Industry and Policymakers

From a managerial perspective, companies in the cultivated meat sector must prioritize addressing consumer concerns through transparent and educational communication. It will be essential to provide clear, accessible information regarding the safety, sustainability, and ethical implications of the production process. Given the strong cultural preference for traditional meat sources in Italy, marketing strategies should position cultivated meat as a complementary product rather than a direct substitute. A gradual introduction could be a more effective approach to facilitate its market entry and eventual adoption.

The establishment of clear, supportive regulatory frameworks that address safety and ethical concerns, alongside targeted incentives for industry development, is vital for building and maintaining consumer confidence. Furthermore, public awareness campaigns and educational initiatives aimed at reducing skepticism and enhancing knowledge could be pivotal in fostering a more informed discourse on alternative proteins.

### 4.2. Limitations and Future Research

This study is not without limitations. First, the sample was limited to a specific geographical context, which may limit the generalizability of the findings to other regions with differing cultural or regulatory landscapes. Second, the cross-sectional design captures attitudes at a single point in time and cannot account for how perceptions and behaviors might evolve. Third, while effective in isolating key drivers, the logistic regression model may have overlooked other potentially relevant variables.

Future research should, therefore, expand on these findings by employing longitudinal studies, considering a wider range of sociodemographic contexts, and exploring deeper attitudinal and ethical dimensions.

## 5. Conclusions

This study explored Italian meat consumer perceptions, knowledge, and willingness to purchase cultivated meat, focusing on the psychological, demographic, and social factors influencing behavior. The results underscore a complex interplay between consumer perceptions, knowledge, and purchasing behavior concerning cultivated meat. A significant knowledge gap among consumers emerged as a critical barrier to its adoption. Psychological factors, including food neophobia and perceived risks, played a more substantial role than sociodemographic variables in shaping attitudes. While environmental and ethical benefits were broadly recognized, safety and cultural identity concerns hindered broader acceptance. Despite these insights, the study highlights critical challenges for introducing cultivated meat into established consumption patterns. Traditional food habits and reliance on conventional meat sources may limit its adoption, suggesting that cultivated meat should be positioned as a complementary product rather than a direct substitute.

## Figures and Tables

**Table 1:** Descriptive statistics of the sample (*n* = 437).

Variable	Mean (Std. Dev.)	Freq. (%)	Min.	Max.
**Age**	33.74 (13.02)		18	86
**Gender**	0.50 (0.50)		0	1
Male		49.66		
Female		50.34		
**Household size**	3.66 (1.06)		1	6
**Living area (Italy)**				
North		11.47		
Center		3.89		
South and Islands		84.67		
**Job**				
Housewife/husband		2.07		
Employee		44.63		
Self-employed		13.97		
Unemployed		2.97		
Pensioner		1.14		
Student		32.95		
**Education**	2.737 (0.973)		1	5
Primary and secondary school		3.66		
High school		40.73		
University degree		45.08		
Master’s and/or PhD		10.53		
**Income**	1.25 (1.01)		0	4
Up to EUR 15,000		21.97		
EUR 15,000–30,000		46.68		
EUR 31,000–45,000		20.37		
EUR 46,000–60,000		6.64		
Over EUR 60,000		4.35		

**Table 2:** Meat consumption habits.

Variable	Frequency (%)
**Types of meat consumed (multiple choice)**	
Beef	95.40
Pork	87.40
Poultry	41.20
Sheep/goat	31.80
Rabbit	9.60
Horse	7.60
Donkey	1.80
**Frequency of meat consumption**	
Less than 2 portions per week	17.20
About 2 portions per week	40.70
More than 2 portions per week	42.10
**Source of meat purchase**	
Butcher shops	58.58
Supermarkets/hypermarkets/discount stores	38.90
Organic stores	0.92
Direct from farmers	0.92
Home farming	0.69

**Table 3:** Awareness and knowledge of cultivated meat.

Variable	Frequency (%)
**Knowledge of cultivated meat**	
Yes	81.92
No	18.08
**Knowledge of production methods**	
Unaware	46.00
Aware and confident in knowledge	8.24
Aware but not confident	45.77

**Table 4:** Willingness to purchase cultivated meat and motivations.

Variable	Frequency (%)
**Willingness to purchase cultivated meat**	
Yes	35.47
No	64.53
**Reasons for interest in cultivated meat**	(Among 155 respondents)
Belief in quality	6.56
Positive perception of synthetic foods	2.63
Environmental sustainability	54.61
Innovation-seeking behavior	25.00
Other	11.20
**Reasons for lack of interest**	(Among 282 respondents)
Health concerns	31.58
Doubts about production processes	34.59
Distrust of new technologies	3.38
Perceived high costs	2.26
Skepticism about environmental impact	6.02
Belief that it is unnatural for humans	15.04
Other	7.13

**Table 5:** Results of the logistic regression.

Purchase	Coef.	St. Err.	*t*-Value	*p*-Value	[95% Conf.	Interval]
**AFTN**	−1.282	0.146	−8.79	*p* < 0.001	−1.568	−0.996
**GCV**	0.529	0.15	3.52	*p* < 0.001	0.235	0.823
HC	−0.058	0.133	−0.43	0.665	−0.318	0.203
EFCM	0.183	0.127	1.44	0.149	−0.066	0.431
CNS	0.19	0.121	1.56	0.118	−0.048	0.427
**Age**	−0.068	0.013	−5.13	*p* < 0.001	−0.094	−0.042
**Gender**	−0.589	0.275	−2.14	0.032	−1.128	−0.051
**Household size**	−0.255	0.128	−1.99	0.047	−0.507	−0.004
Education	0.061	0.137	0.44	0.657	−0.207	0.328
**Income**	0.361	0.128	2.82	0.005	0.11	0.612
**Cultivated meat awareness**	0.713	0.394	1.81	0.07	−0.06	1.486
Constant	2.01	0.961	2.09	0.036	0.127	3.893
Mean dependent var.	0.355		SD dependent var.		0.479	
Pseudo-r-squared	0.352		Number of obs.		437	
Chi-squared	200.133		Prob. > chi2		0.000	
Akaike crit. (AIC)	392.231		Bayesian crit. (BIC)		441.190	

**Table 6:** Results of the logistic regression (odds ratio).

Purchase	Odds Ratio	St. Err.	*t*-Value	*p*-Value	[95% Conf.	Interval]
**AFTN**	0.277	0.04	−8.79	*p* < 0.001	0.208	0.369
**GCV**	1.697	0.255	3.52	*p* < 0.001	1.265	2.278
HC	0.944	0.126	−0.43	0.665	0.727	1.225
EFCM	1.201	0.152	1.44	0.149	0.936	1.539
CNS	1.209	0.146	1.56	0.118	0.953	1.533
**Age**	0.935	0.012	−5.13	*p* < 0.001	0.911	0.959
**Gender**	0.555	0.152	−2.14	0.032	0.324	0.95
**Household size**	0.775	0.1	−1.99	0.047	0.602	0.996
Education	1.063	0.145	0.44	0.657	0.813	1.389
**Income**	1.434	0.184	2.82	0.005	1.116	1.843
**Cultivated meat awareness**	2.041	0.805	1.81	0.07	0.942	4.42
Constant	7.462	7.17	2.09	0.036	1.135	49.057
Mean dependent var.	0.355		SD dependent var.		0.479	
Pseudo-r-squared	0.352		Number of obs.		437	
Chi-squared	200.133		Prob. > chi2		0.000	
Akaike crit. (AIC)	392.231		Bayesian crit. (BIC)		441.190	

## Data Availability

The dataset is available upon request from the authors. The datasets are not publicly available due to time limitations.

## Abbreviations

The following abbreviations were used in this manuscript:

AFTN	Abbreviated Food Technology Neophobia
GCV	Green Consumer Value
HC	Health Consciousness
EFCM	Ethical Food Choice Motives
CNS	Consumer Novelty Seeking
OR	Odds Ratio

## References

[1] (2024). United Nations. World Population Prospects 2024: Summary of Results. https://population.un.org/wpp/.

[2] FAO (2020). The State of Food Security and Nutrition in the World 2020.

[3] Gu D., Andreev K., Dupre M.E. (2021). Major trends in population growth around the world. China CDC Wkly..

[4] Chodkowska K.A., Wódz K., Wojciechowski J. (2022). Sustainable future protein foods: The challenges and the future of cultivated meat. Foods.

[5] Ritchie H., Rosado P., Roser M. (2019). Meat and Dairy Production.

[6] Chen L., Guttieres D., Koenigsberg A., Barone P.W., Sinskey A.J., Springs S.L. (2022). Large-scale cultured meat production: Trends, challenges and promising biomanufacturing technologies. Biomaterials.

[7] Sakadevan K., Nguyen M.L. (2017). Livestock production and its impact on nutrient pollution and greenhouse gas emissions. Adv. Agron..

[8] Burlingame B., Moltedo A., Cafiero C. (2024). Global protein sustainability and the United Nations, through to the 2030 agenda. Front. Nutr..

[9] Nirmal N., Anyimadu C.F., Khanashyam A.C., Bekhit A.E.D.A., Dhar B.K. (2024). Alternative Protein Sources: Addressing Global Food Security and Environmental Sustainability. Sustain. Dev..

[10] Pajčin I., Knežić T., Savic Azoulay I., Vlajkov V., Djisalov M., Janjušević L., Grahovac J., Gadjanski I. (2022). Bioengineering Outlook on Cultivated Meat Production. Micromachines.

[11] Habowski K., Sant’Ana A.S. (2024). Microbiology of cultivated meat: What do we know and need to know?. Trends Food Sci. Technol..

[12] Vural Gursel I., Sturme M., Hugenholtz J., Bruins M. (2022). Review and Analysis of Studies on Sustainability of Cultured Meat.

[13] Foran L., Bauks J. (2023). The Potential of Cell and Plant-Based Meat Alternatives to Improve Global Health Outcomes. J. Stud. Res..

[14] Munteanu C., Mireşan V., Răducu C., Ihuţ A., Uiuiu P., Pop D., Neac A., Cenariu M., Groza I. (2021). Can cultured meat be an alternative to farm animal production for a sustainable and healthier lifestyle?. Front. Nutr..

[15] Rasmussen M.K., Gold J., Kaiser M.W., Moritz J., Räty N., Rønning S.B., Ryynänen T., Skrivergaard S., Ström A., Therkildsen M. (2024). Critical review of cultivated meat from a Nordic perspective. Trends Food Sci. Technol..

[16] Tuomisto H.L., Ryynänen T. (2024). Environmental Impacts of Cultivated Meat. Cultivated Meat: Technologies, Commercialization and Challenges.

[17] Nobre F.S. (2022). Cultured meat and the sustainable development goals. Trends Food Sci. Technol..

[18] Treich N. (2021). Cultured meat: Promises and challenges. Environ. Resour. Econ..

[19] Da Silva I.M. (2024). EU DECODED: Could Lab-Grown Meat Arrive in Supermarkets Soon?.

[20] European Commission (2015). Regulation (EU) 2015/2283 of the European Parliament and of the Council of 25 November 2015 on novel foods, amending Regulation (EU) No 1169/2011 of the European Parliament and of the Council and amending repeal Regulation (EC) No 258/97 of the European Parliament and of the Council and Regulation (EC) No 1852/2001 of the Commission. https://eur-lex.europa.eu/eli/reg/2015/2283/oj/eng.

[21] Mancini M.C., Antonioli F. (2022). Italian consumers standing at the crossroads of alternative protein sources: Cultivated meat, insect-based and novel plant-based foods. Meat Sci..

[22] Di Vita G., Maesano G., Zanchini R., Barbieri C., Spina D., Caracciolo F., D’Amico M. (2022). The thin line between tradition and well-being: Consumer responds to health and typicality attributes for dry-cured ham. J. Clean. Prod..

[23] Uliano A., Stanco M., Lerro M. (2024). Perception is not reality: Uncovering the adherence to the Mediterranean diet. J. Agric. Food Res..

[24] Nazzaro C., Stanco M., Uliano A., Marotta G. (2024). Consumers’ acceptance and willingness to pay for enriched foods: Evidence from a choice experiment in Italy. Future Foods.

[25] Faccio E., Guiotto Nai Fovino L. (2019). Food neophobia or distrust of novelties? Exploring consumers’ attitudes toward GMOs, insects and cultured meat. Appl. Sci..

[26] Baum C.M., Bröring S., Lagerkvist C.J. (2021). Information, attitudes, and consumer evaluations of cultivated meat. Food Qual. Prefer..

[27] Szejda K., Stumpe M., Raal L., Tapscott C.E. (2021). South African consumer adoption of plant-based and cultivated meat: A segmentation study. Front. Sustain. Food Syst..

[28] Heijnk V., Espey A., Schuenemann F. (2023). A comparison of influencing factors on attitudes towards plant-based, insect-based and cultured meat alternatives in Germany. Food Qual. Prefer..

[29] Engel L., Vilhelmsen K., Richter I., Moritz J., Ryynänen T., Young J.F., Burton R.J.F., Kidmose U., Klöckner C.A. (2024). Psychological factors influencing consumer intentions to consume cultured meat, fish and dairy. Appetite.

[30] Ramlo S. (2011). Using word clouds to visually present Q methodology data and findings. J. Hum. Subj..

[31] Schnettler B., Grunert K.G., Miranda-Zapata E., Orellana L., Sepúlveda J., Lobos G., Hueche C., Höger Y. (2017). Testing the Abbreviated Food Technology Neophobia Scale and its relation to satisfaction with food-related life in university students. Food Res. Int..

[32] Taufique K.M.R., Siwar C.B., Talib B.A., Chamhuri N. (2014). Measuring consumers’ environmental responsibility: A synthesis of constructs and measurement scale items. Curr. World Environ..

[33] Gould S.J. (1988). Consumer attitudes toward health and health care: A differential perspective. J. Consum. Aff..

[34] Lindeman M., Väänänen M. (2000). Measurement of ethical food choice motives. Appetite.

[35] Manning K.C., Bearden W.O., Madden T.J. (1995). Consumer innovativeness and the adoption process. J. Consum. Psychol..

[36] Tanujaya B., Prahmana R.C.I., Mumu J. (2022). Likert scale in social sciences research: Problems and difficulties. FWU J. Soc. Sci..

[37] Rojas-Rivas E., Espinoza-Ortega A., Thomé-Ortiz H., Cuffia F. (2022). More than words! A narrative review of the use of the projective technique of word association in the studies of food consumer behavior: Methodological and theoretical implications. Food Res. Int..

[38] Freire C.E.C.D.A., Patinho I., Gonçalves S.F., Cançado M.P., Saldaña E., de Alencar S.M., Cesar A.S.M. (2024). Utilising free comments and textual analysis to identify knowledge and acceptance of functional dairy products: A Brazilian perspective. Int. J. Dairy Technol..

[39] Liang A.R.D., Yang W., Chen D.J., Chung Y.F. (2017). The effect of sales promotions on consumers’ organic food response: An application of logistic regression model. Br. Food J..

[40] Uliano A., Stanco M., Lerro M., Marotta G., Nazzaro C. (2022). Evaluating citizen-consumers’ attitude toward high social content products: The case of social farming. Br. Food J..

[41] De Devitiis B., Bimbo F., Viscecchia R., Nardone G., Seccia A., Monacis L., Albenzio M., Santillo A. (2023). Consumer acceptance for sheep milk–based yogurt—Evidence from a large sample of Italian consumers. J. Dairy Sci..

[42] Van der Stricht H., Hung Y., Fischer A.R., Verbeke W. (2024). Consumer segments less or more willing to adopt foods with microalgae proteins. Food Qual. Prefer..

[43] Uliano A., Stanco M., Marotta G., Nazzaro C. (2024). Combining healthiness and sustainability: An analysis of consumers’ preferences and willingness to pay for functional and sustainable snack bars. Future Foods.

[44] Halvorson M.A., McCabe C.J., Kim D.S., Cao X., King K.M. (2022). Making sense of some odd ratios: A tutorial and improvements to present practices in reporting and visualizing quantities of interest for binary and count outcome models. Psychol. Addict. Behav..

[45] Dalle Zotte A., Brugiapaglia A., Cullere M. (2017). What is meat in Italy?. Anim. Front..

[46] Farchi S., De Sario M., Lapucci E., Davoli M., Michelozzi P. (2017). Meat consumption reduction in Italian regions: Health co-benefits and decreases in GHG emissions. PLoS ONE.

[47] Cocking C., Walton J., Kehoe L., Cashman K.D., Flynn A. (2020). The role of meat in the European diet: Current state of knowledge on dietary recommendations, intakes and contribution to energy and nutrient intakes and status. Nutr. Res. Rev..

[48] González-Azcárate M., Maceín J.L.C., Bardají I. (2021). Why buying directly from producers is a valuable choice? Expanding the scope of short food supply chains in Spain. Sustain. Prod. Consum..

[49] Kantono K., Hamid N., Ma Q., Chadha D., Oey I. (2021). Consumers’ perception and purchase behaviour of meat in China. Meat Sci..

[50] Crovato S., Pinto A., Di Martino G., Mascarello G., Rizzoli V., Marcolin S., Ravarotto L. (2022). Purchasing habits, sustainability perceptions, and welfare concerns of Italian consumers regarding rabbit meat. Foods.

[51] Staudigel M., Trubnikov A. (2022). High price premiums as barriers to organic meat demand? A hedonic analysis considering species, cut and retail outlet. Aust. J. Agric. Resour. Econ..

[52] Meijer G.W., Lähteenmäki L., Stadler R.H., Weiss J. (2021). Issues surrounding consumer trust and acceptance of existing and emerging food processing technologies. Crit. Rev. Food Sci. Nutr..

[53] Siegrist M., Hartmann C. (2020). Consumer acceptance of novel food technologies. Nat. Food.

[54] Siegrist M., Hartmann C. (2020). Perceived naturalness, disgust, trust and food neophobia as predictors of cultured meat acceptance in ten countries. Appetite.

[55] Li L., Wang Z., Li Y., Liao A. (2021). Impacts of consumer innovativeness on the intention to purchase sustainable products. Sustain. Prod. Consum..

[56] Zarba C., Chinnici G., Hamam M., Bracco S., Pecorino B., D’Amico M. (2022). Driving management of novel foods: A network analysis approach. Front. Sustain. Food Syst..

[57] Giacalone D., Jaeger S.R. (2023). Consumer acceptance of novel sustainable food technologies: A multi-country survey. J. Clean. Prod..

[58] Heiskanen A., Ryynänen T. (2024). Optimists, moderates and sceptics–identifying consumer groups and their willingness to consume cultured proteins in Finland. Br. Food J..

[59] Szejda K., Bryant C.J., Urbanovich T. (2021). US and UK consumer adoption of cultivated meat: A segmentation study. Foods.

[60] Nazzaro C., Uliano A., Lerro M., Stanco M. (2025). From claims to choices: How health information shapes consumer decisions in the functional food market. Foods.

[61] Szendrő K. (2025). Consumer perceptions of lab-grown cells: Awareness, barriers, and the power of information. A review. Czech J. Anim. Sci..

[62] Haq M.A., Meghwar P., Maggiolino A. (2025). Consumer Preferences, Safety, and Legislation of Cultured Meat. Innovative Technologies for Meat Processing.

[63] Keefer H., Racette C., Drake M. (2024). Factors influencing consumer motivations for protein choice. J. Food Sci..

[64] Nguyen J., Ferraro C., Sands S., Luxton S. (2022). Alternative protein consumption: A systematic review and future research directions. Int. J. Consum. Stud..

[65] Tso R., Lim A.J., Forde C.G. (2020). A critical appraisal of the evidence supporting consumer motivations for alternative proteins. Foods.

[66] Casalegno C., Candelo E., Santoro G. (2022). Exploring the antecedents of green and sustainable purchase behaviour: A comparison among different generations. Psychol. Mark..

[67] Ivanova O., Flores-Zamora J., Khelladi I., Ivanaj S. (2019). The generational cohort effect in the context of responsible consumption. Manag. Decis..

[68] Casini L., Contini C., Romano C., Scozzafava G. (2015). Changes in dietary preferences: New challenges for sustainability and innovation. J. Chain Netw. Sci..

[69] Vermeir I., Verbeke W. (2008). Sustainable food consumption among young adults in Belgium: Theory of planned behaviour and the role of confidence and values. Ecol. Econ..

[70] Bryant C., Barnett J. (2018). Consumer acceptance of cultured meat: A systematic review. Meat Sci..

[71] Palmieri N., Perito M.A., Lupi C. (2020). Consumer acceptance of cultured meat: Some hints from Italy. Br. Food J..

[72] Weinrich R., Strack M., Neugebauer F. (2020). Consumer acceptance of cultured meat in Germany. Meat Sci..

[73] Fu W., Zhang H., Whaley J.E., Kim Y.K. (2023). Do consumers perceive cultivated meat as a sustainable substitute to conventional Meat? Assessing the facilitators and inhibitors of cultivated meat acceptance. Sustainability.

